# Review of Hydrogen Sensors in Aerobic and Anaerobic Environments Coupled with Artificial Intelligence Tools

**DOI:** 10.3390/s25226936

**Published:** 2025-11-13

**Authors:** Jordan Herbeck-Tazibt, Mohand A. Djeziri, Tomas Fiorido, Jean-Luc Seguin

**Affiliations:** Centre National de la Recherche Scientifique (CNRS) and Institut Matériaux Microélectronique Nanosciences de Provence (IM2NP), Aix-Marseille Université, Université de Toulon, 13397 Marseille, France; mohand.djeziri@im2np.fr (M.A.D.); tomas.fiorido@im2np.fr (T.F.); jean-luc.seguin@im2np.fr (J.-L.S.)

**Keywords:** hydrogen sensors, artificial intelligence, modeling, physics, materials, mathematical algorithm

## Abstract

**Highlights:**

This review highlights the potential applications of hydrogen gas, with a focus on specific sensor types for aerobic and anaerobic applications.

**What are the main findings?**

- This article provides a comprehensive and recent survey of intelligent and resistive hydrogen sensors according to type of application, in comparison with other sensor technologies, and identifies those coupled with artificial intelligence, detailing the methods and algorithms used, and identifying those in place within sensors in the literature.
- In the literature, very few studies deal jointly with aerobic and anaerobic hydrogen applications.
- Existing hydrogen sensor technologies in the literature are listed and compared according to potential hydrogen applications.
- This review shows the types of hydrogen sensors coupled with a software layer.

**What are the implication of the main findings?**

- Each field of application is matched to the type of sensor used, with explicit detection ranges based on hydrogen-specific standards and regulations.
- In this paper, the use of Artificial Intelligence within aerobic and anaerobic hydrogen sensors is necessary for performance improvement.
- The main methods and algorithms used in hydrogen sensors, from the simplest to the most complex, are discussed and compared with sensors coupled with AI tools. Their performance is also assessed.

**Abstract:**

Hydrogen-based technologies are progressing in several areas, such as transportation and energy, especially regarding their use as a replacement for greenhouse gas-emitting fuels. However, hydrogen is known for its explosiveness and large-scale flammability; hence, there is a need to ensure it can be detected and measured without risk. Several types of hydrogen sensors are available on the market. Each sensor is suited to a specific environment and operating conditions. In recent years, Artificial Intelligence tools have been increasingly used to improve the design and performance of these sensors in terms of safety, reliability, sensitivity, speed, and selectivity. This paper provides a review of available hydrogen sensors, their fields of application, and the main directions explored by the scientific community for integrating Artificial Intelligence tools to improve their performance. A comparative analysis is presented based on criteria related to sensor technologies, data processing tools, and target performance. This review highlights the results achieved and the challenges that remain to be addressed in various application fields.

## 1. Introduction

Hydrogen is a promising multi-purpose energy carrier with a wide range of applications. The International Energy Agency (IEA) consider hydrogen as a major technological, economic, and environmental challenge [1], and admits that its energy properties are environmentally friendly [2]. It is increasingly used in many fields, such as clean energy and sustainable mobility [3]; chemical and metallurgical industry; transportation systems (trains, airplanes, buses, cars) [1,4]; medicine, for its antioxidant properties; and environmental protection for water treatment and pollution control [5].

However, hydrogen is a colorless, odorless, and highly volatile gas, with a great capacity to leak from its containers. It is also highly flammable, with a flammability and explosivity range in air ranging from 4% to 75%, which requires reliable tools for hydrogen detection in real-time measurement, accurately and at an early stage [6].

To meet the demands of hydrogen gas control and monitoring, numerous sensors have been designed and tested according to the requirements and standards relating to hydrogen use and exposure [6]. They are based on operating principles specific to each application field [7]. In addition, Artificial Intelligence is used to improve sensor design [8] and performance, such as sensitivity, stability, selectivity, detection kinetics, concentration ranges, and robustness to interferents [9].

A significant amount of research is devoted to intelligent hydrogen sensors, such as the design of porous silicon hydrogen sensors [10], based on SnO_2_ ([11,12,13]), Pd or alloys Pd-M (M = Ni, Ag, Au) [14], other semiconductor materials such as ZnO, WO_3_, and In_2_O_3_ [15], or nanostructured composites [16]. These sensors are designed for specific infrastructures [17], stand-alone devices [18,19], or for a wide variety of applications [20]. However, to the best of our knowledge, there is no comprehensive review of hydrogen sensors in aerobic and anaerobic environments, including an analysis of AI’s contribution to improving their performance. This review paper details the state-of-the-art of hydrogen sensors in aerobic and anaerobic environments, as well as the contributions of Artificial Intelligence in improving their performance in different application domains. This fills the gap by offering a structured mapping of existing technologies and recent advances.

This paper is organized as follows. Section 2 covers standards and applications using hydrogen sensors in anaerobic and aerobic environments. In Section 3, a study of hydrogen sensors coupled with artificial intelligence tools is carried out, with the measurements made, the performance obtained, and the methods and algorithms aimed at achieving target performance in sensor improvement. An analysis and discussion are provided in Section 3. A conclusion is presented in Section 4.

## 2. Standards and Applications Using Hydrogen Sensors

A wide variety of sensors based on different principles and materials are available, including optical, electrochemical, catalytic, electrical, thermal conductivity, resistive, metal film, and semiconductor sensors. There are two main areas of application for hydrogen sensors: the anaerobic environment (without oxygen), and the aerobic environment (presence of oxygen and/or humidity). Each environment presents specific standards that impact the design and performance of the sensors.

### 2.1. Standards

Maximum exposure values and detection limits for hydrogen must be known for every application and for every use of a hydrogen sensor [21]. These values are based on industry standards and recommendations for the management of explosion risks and human health. According to the Occupational Safety and Health Administration (OSHA) [22], hydrogen parameters are essential for correctly monitoring systems and ensuring safety in installations. These values are presented in Table 1. They are supported by the French National Institute for Research and Safety (INRS) [23,24], and meet the following requirements:

- ISO/TR 15916 [25]: Safety in hydrogen systems.
- ATEX (EXplosive ATmospheres) [26]: Standards for high-risk environments.
- European Directive SEVESO III [27]: Industrial risk management.
- OSHA and NIOSH [28]: Work environment regulations.

**Table 1:** Exposure limits and detection values recommended by OSHA [22] and INRS [23,24], justified by the Safety Data Sheets (SDS) [26,29].

**Oxygen level in case of leak**	<19.5%
**Lower Explosive Limit (LEL)**	4%
**Upper Explosive Limit (UEL)**	75%
**Average Exposure Limit (AEL)**	8 h (1000 ppm)
**Short Term Exposure Limit (STEL)**	15 min (1000 ppm)
**Sensor Detection Limit (SDL)**	0.1 ppm–100% in volume (environment)
**Recommended sensitivity range**	0.1 ppm–1% is often sufficient for Detect leaks early. Prevent the concentration from reaching the LEL (4% vol).

### 2.2. Hydrogen Sensors

Numerous hydrogen sensors based on different materials are being studied [30]. They are grouped into families by type of technology, and each targets a specific application.

**Figure 1:**
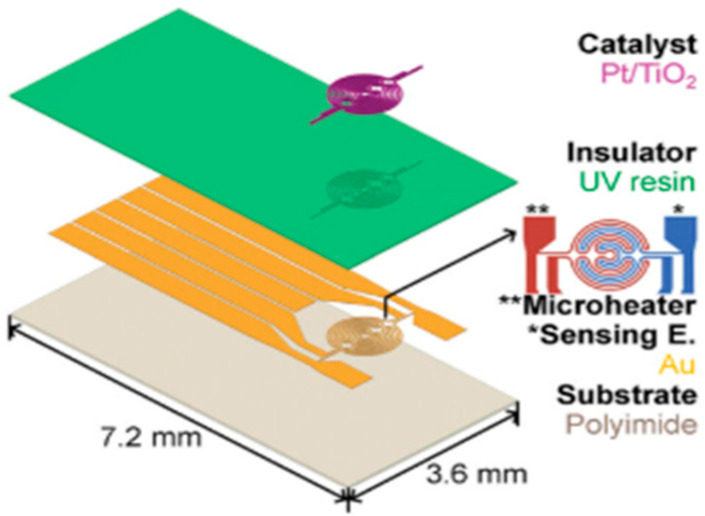
Physical principle of a catalytic sensor. The geometric dimensions of the channels are 14 µm deep and 20–30 µm wide, which are aligned with the microheater ** and sensing electrode *.

Catalytic sensors [31,32,33] are used for hydrogen detection. They work by oxidizing hydrogen on a catalytic surface (often platinum), which generates heat (an exothermic reaction). This heat increases the temperature of a sensitive element (such as a heating wire), which modifies its electrical resistance, hence the signal measurement. They are simple and robust, but require oxygen for operation and are highly sensitive to interferents. An example of a catalytic sensor is shown in Figure 1. It consists of a substrate, a heating element and a sensitive track, and the catalytic surface is based on Pt/TiO_2_ [32].

**Figure 2:**
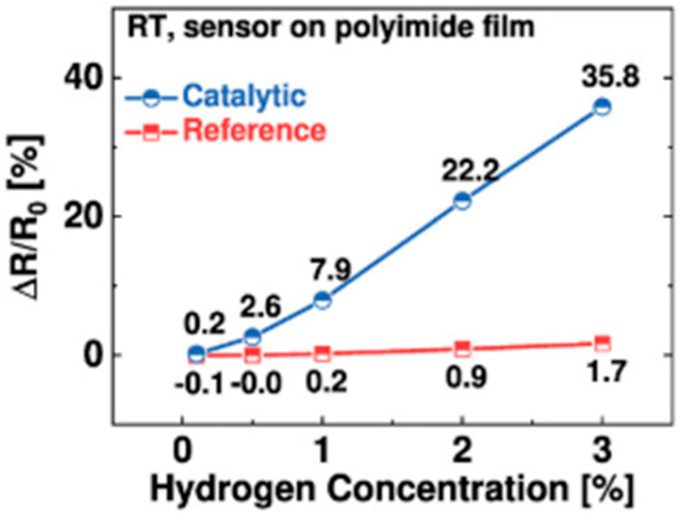
Response of a catalytic sensor as a function of H_2_ concentration and comparison with a reference sensor on polyimide film.

The response of this sensor to hydrogen and a comparison with a reference sensor are illustrated in Figure 2.

Figure 2 shows that, under 3% H_2_ exposure, the catalytic sensor has responses in excess of 30%, while the reference sensor has responses of the order of 1.7%. The Pt/TiO_2_-based catalytic sensor is therefore more sensitive to hydrogen than a reference sensor on polyimide film [32].

**Figure 3:**
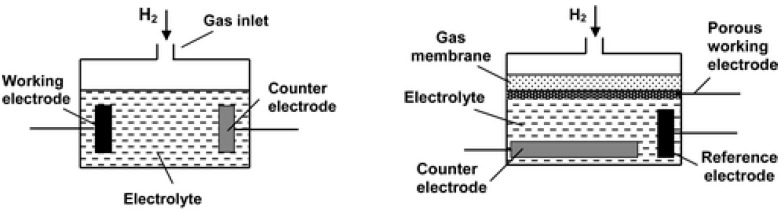
Schematic of two-electrode (**left**) and three-electrode (**right**) electrochemical sensors [34].

Electrochemical sensors [33,34,35] consist of a sensing electrode and a counter-electrode, separated by a thin layer of electrolyte. Their detection principle is based on an electrochemical reaction between hydrogen gas and the electrode, resulting in a measurable variation in current (amperometric sensors) or electrical potential (potentiometric sensors). Amperometric sensors generate an electric current proportional to the hydrogen concentration, while potentiometric sensors measure the voltage variation associated with the chemical reaction [36]. These sensors have good accuracy but use an electrolyte with a limited service life [7]. The operating principle of an amperometric electrochemical sensor is illustrated in Figure 3.

**Figure 4:**
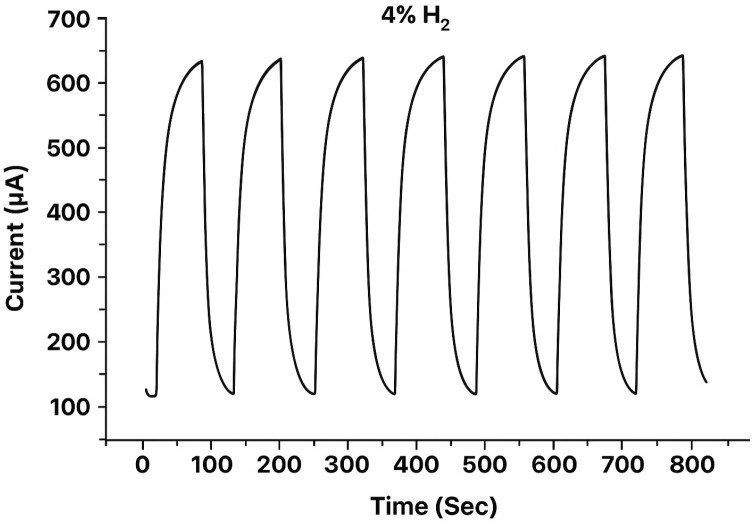
Cyclic behavior of a Pt-based electrochemical sensor exposed to 4% H_2_ [34].

An example of the response of this type of sensor at 4% H_2_ is shown in Figure 4. Good measurement repeatability and baseline stability are observed.

**Figure 5:**
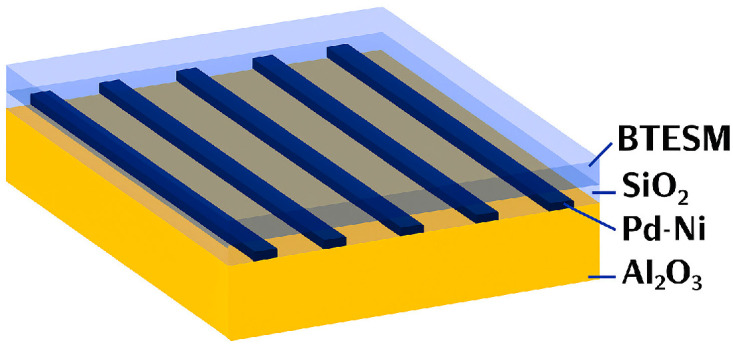
Diagram of a Si-Pd-Ni MOX sensor.

**Figure 6:**
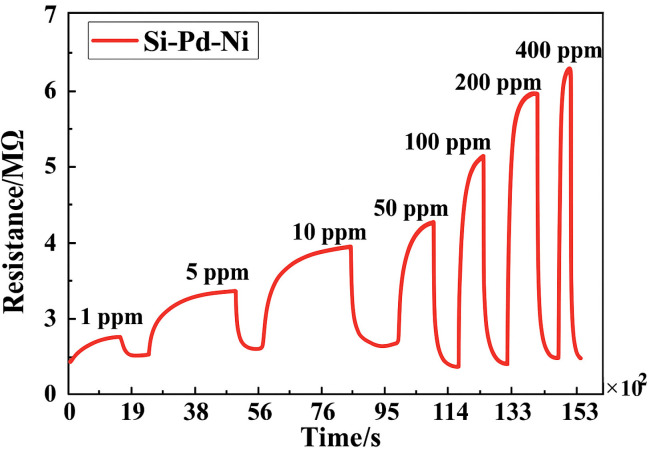
Exposure of MOX sensor to low concentrations of H_2_.

**Semiconductor metal oxide sensors (MOX)** are manufactured from materials such as tin dioxide (SnO_2_), zinc oxide (ZnO), or titanium dioxide (TiO_2_), usually integrated as thin layers or beads in a porous ceramic matrix around a heating coil [37,38,39,40,41]. The operation of these sensors requires a high temperature, typically between 200 °C and 400 °C, in order to activate chemical reactions on the oxide surface and achieve stable, measurable conductivity. The detection principle is based on the variation in the electrical conductivity of the semiconductor material in the presence of hydrogen. At high temperatures, oxygen adsorbed on the surface captures electrons, forming a depleted layer characterized by high resistance. When a hydrogen-containing atmosphere is present, it reacts with the adsorbed oxygen, releasing electrons into the material and causing a drop in electrical resistance. This variation is directly correlated to hydrogen concentration [16,37,40]. They can be influenced by temperature and humidity [12]. They are small, inexpensive to manufacture, and sensitive to hydrogen, and have a fast response time [37]. An example of a MOX sensor is shown in Figure 5 [40]. This is a ternary Si-Pd-Ni alloy sensor with a chemical treatment (BTESM) on a Al_2_O_3_ substrate. This sensor is sensitive to hydrogen and detects low concentrations from 1 ppm to 400 ppm, as shown in Figure 6 [40]. However, the sensor’s stability is poor at baseline and with exposure. These performances can be improved by using a software layer, as proposed in [37].

**Thermal conductivity sensors** [37] can detect large hydrogen concentrations of up to 100%, with a response and return time of just a few seconds. These sensors exploit the difference in thermal conductivity between hydrogen and other gases to measure its concentration. They generally use a heating probe whose temperature or resistance varies according to the heat transfer in the surrounding gas. Hydrogen has a different thermal conductivity to other gases. They work well at low humidity and are not poisoned by other gases, but they are less accurate at low hydrogen concentrations [38].

**Figure 7:**
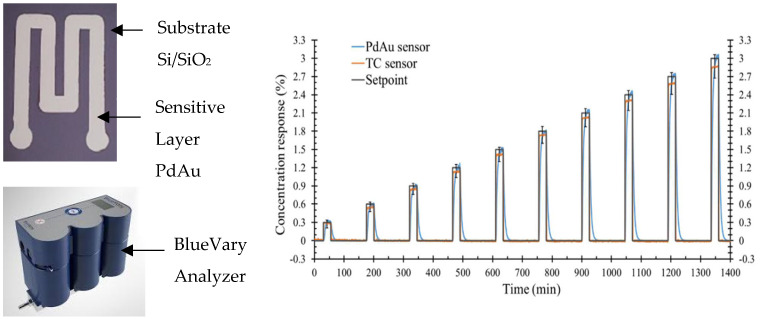
PdAu macrosensor and BlueVary analyzer (**left**); concentration response profile of a PdAu sensor and a commercial thermal conductivity sensor for stepwise exposure (**right**) [42,45].

Metal film sensors [42,43] have a detection principle based on the measurement of variations in their physical properties through the reversible interaction of the layer with hydrogen. Palladium absorbs hydrogen to form a metal hydride, resulting in a change in physical properties and a variation in resistance. They are highly selective to hydrogen and sensitive, especially in Pd and Pt, but have long response times and significant aging [42,43,44]. A macro-sensor on a Si/SiO_2_ substrate was produced, and a PdAu alloy layer was deposited by RF sputtering. This sensor is exposed to hydrogen concentrations ranging from 0.3% to 3% and a comparison is made with a commercial thermal conductivity sensor in Figure 7.

By comparative standards, both sensors offer optimum stability, reliability and sensitivity to hydrogen. Nevertheless, the PdAu macro-sensor exhibits higher responses than a commercial analyzer-type sensor, particularly for high hydrogen concentrations. Performance is better with a macro-sensor. Furthermore, this device enables fast, real-time measurements, and is relatively less expensive than an analyzer, where measurements are spotty and sometimes time-consuming.

**Figure 8:**
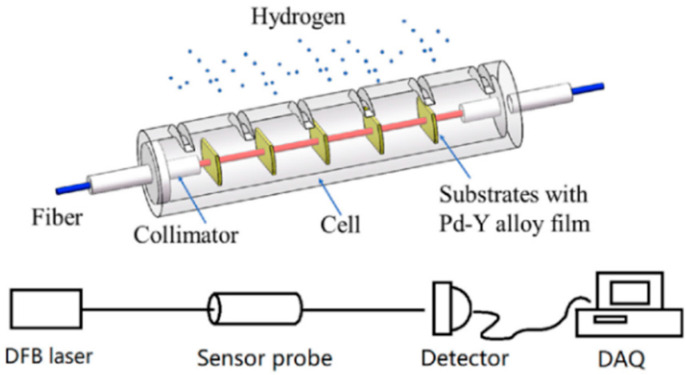
Fiber optic hydrogen sensor.

Spectroscopic [46,47] and fiber-optic sensors [48,49] where hydrogen-sensitive layers change their optical properties in the presence of hydrogen, are promising for its detection. They are highly sensitive, unaffected by electrical or magnetic interference, and suitable for complex environments. Fibers are generally made of silica, but PdNi and PdAg alloys are sometimes used [50]. However, they are sensitive to temperature variations and interferents such as H_2_S and NH_3_, and are much more expensive. They are often useful for monitoring hydrogen in environments where electrical safety is critical [50]. An example of a transmission fiber-optic hydrogen sensor with multilayer Pd-Y alloy films is presented in Figure 8. Its probe consists of a cell, a pair of collimators, and several films with palladium alloy nanofilms. On either side of the gas cell, two collimators are fixed and aligned. A series of Pd/Y alloy film substrates of equal thickness are parallel and fixed in the cell. Light from a laser diode penetrates all the aligned films through the collimators. When hydrogen gas is injected into the cell, all the thin films can interact simultaneously with the light and the hydrogen [47].

**Figure 9:**
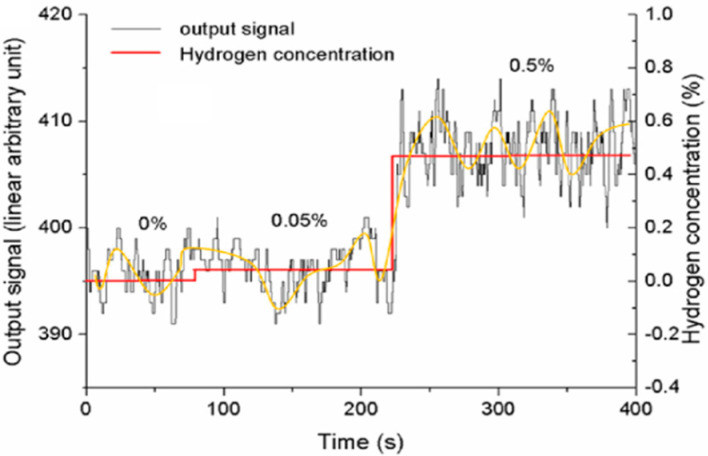
Sensor response at 0%, 0.05%, and 0.5% H_2_.

In Figure 9, we can see that the noise is greater than the signal at a concentration of 0.05% H_2_. This shows that the sensor cannot recognize a concentration below 0.05% H_2_. However, it does provide responses at 0.5% H_2_. Spectral analysis and signal processing can contribute to sensor stability and drift [47].

### 2.3. Anaerobic Applications

Since the famous hydrogen disaster linked to the Hindenburg Fire on 6 May 1937, most recent scientific work has focused on measuring hydrogen to monitor and prevent leaks of hydrogen or combustible gas into the air. Operating in an anaerobic environment was given very little attention. With recent technological advances and the desire to anchor the energy transition in the collective memory and in the world, the anaerobic environment has become increasingly important, particularly in the transport sector, with hydrogen-powered vehicles, trains, buses, aircraft, and even rockets and streetcars [7].

#### 2.3.1. Power to Gas (P2G)

**Figure 10:**
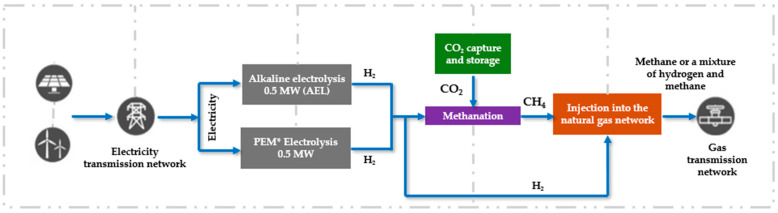
Illustration of Power-to-Gas [52].

Power-to-Gas establishes a bridge between electricity and gas networks. The energy surplus is used to perform water electrolysis via electrolyzers (Alkaline Electrolyzer (AEL) & Proton Exchange Membrane (PEM*)) to produce green hydrogen. This hydrogen is then either directly injected into the natural gas network or used in a methanation reaction (Sabatier reaction) with CO_2_ captured from industrial sites or recovered from biogas. The resulting synthetic gas can be stored for extended periods and used to offset electricity production deficits through fuel cells or gas turbines. An illustration of the Power-to-Gas principle and its applications is presented in Figure 10. Power-to-Gas technology thus helps reduce the need to balance the electricity grid [51].

When injecting hydrogen into the natural gas transmission network, it is essential to monitor and control its concentration in real-time; hence it is important to use a sensor that can measure the different levels of hydrogen.

This technology is based on a concentration range from 1 to 20% dihydrogen, with a pressure range of around 30 bars. The interferents for this type of application are carbon monoxide (CO) and dihydrogen sulfide (H_2_S) [51].

The sensors used for this application must guarantee optimum hydrogen detection in anaerobic conditions, in a dry environment, below 100 °C, in a wide concentration range from 0 to 100%, and must be selective to hydrogen, with a response and return time of less than one minute [42,45,51,52].

**Catalytic sensors** are not suitable for measuring hydrogen in anaerobic environments and high-concentration zones, as they require the presence of oxygen for an oxidation reaction that leads to hydrogen detection [52].

**Electrochemical sensors** are used in anaerobic environments, but they only work in humid environments, as hydrogen detection relies on ambient humidity of between 20% and 60% (absent from gas lines), which will lead to a loss of resistance and subsequent sensor degradation [53].

**Thermal conductivity sensors** are highly sensitive to variations in several components with different thermal conductivities, resulting in baseline drift. They are therefore not selective to hydrogen [54].

**MOX sensors,** where the presence of oxygen is mandatory to enable load variation, are not suitable. This sensor technology cannot operate in an anaerobic environment, but can operate in an aerobic environment [55].

**Metal-film sensors** appear to be the most suitable for this type of application [42,52].

As far as Power to Gas is concerned, European discussions on maximum injection rates and consumer requirements in terms of sensitivity and detection speed are still ongoing [51], but the specifications shown in Table 2 are an illustration of the basic requirements for this type of application:

A Pd film-based sensor can repeatedly detect different hydrogen concentrations in aerobic or anaerobic environments at room temperature [42]. However, the repeated exposure of pure Pd to hydrogen causes problems: high measurement hysteresis, long response and feedback times, loss of detection properties, etc. [56]. Optimization of the film’s physico-chemical properties is possible to overcome these problems. [42].

Other sources [48,57,58] assert the use of optical hydrogen sensors in which hydrogen interacts with an optical fiber coated with a sensitive material, modifying light transmission or reflection. Optical hydrogen sensors with reflexivity and transmission [48,58] and surface plasmon resonance [57] are reliable in terms of selectivity and sensitivity to hydrogen.

#### 2.3.2. Monitoring Tanks on Future Vehicles

Monitoring the fuel tanks of future vehicles is one of the most important applications for hydrogen. To monitor hydrogen tanks in cars, planes, trains, buses, streetcars, and rockets, sensors must meet stringent requirements in terms of safety, accuracy, durability, and environmental compatibility [59,60]. Criteria such as sensitivity and accuracy must be respected in order to detect minute concentrations before the gas reaches the Lower Explosive Limit (LEL). Other criteria, such as resistance to repeated charging and discharging cycles, variations in temperature, pressure and humidity, and low energy consumption, are also essential [59]. Similarly, robustness against vibration and electromagnetic interference must be guaranteed [59]. Depending on the type of vehicle, specific technologies will be chosen to meet the specific constraints of the operating environment (high pressure for trains, low weight for aircraft, and low cost for cars) [60].

The recent review article by Lee, Seoung-Ki Lee [61] and their colleagues discusses the use of hydrogen sensors for mobility and transport infrastructure. For each type of sensor, response and return times, hydrogen concentration ranges, operating temperatures, sensitivity values, and detection materials were studied and listed for tank monitoring in future vehicles.

According to the Occupational Safety and Health Administration (OSHA) [22,28] and the National Institute for Research and Safety (INRS) [23,26,29], detection limits are different for each vehicle, and it is necessary to adapt the type of sensor to the range, from 10 ppm to 4% for aircraft, 100 ppm to 4% for trains, buses and cars, and 10 ppm to 100% for high-pressure tanks [62,63]. Table 3 uses recent data [61] to summarize these studies.

Hydrogen sensors are available and adapted to the targeted concentrations depending on the type of vehicle. Detection ranges are specific and known for these sensors, as shown in Table 4.

Based on the works of [60,61,64], we can see the following.

**Semiconductor sensors** based on materials such as SnO_2_ and ZnO, with a measurement range from 1 ppm to 1%, are sensitive to low concentrations. They are relatively inexpensive, compact, and lightweight, and can detect leaks close to vehicle fuel tanks. However, they are likely to interfere with other gases, such as CO and CH_4_, and are sensitive to the influence of temperature (below 200 °C) and humidity (20–100% RH) [60,61].

**Electrochemical sensors** based on platinum, palladium, aluminum, nickel, or cobalt, operate over a wide range from 10 ppm to 10% and are precise and reliable for critical applications. They are used in detection systems and are sensitive and selective to hydrogen, guaranteeing low energy consumption, but have a limited lifetime and operate only under humidity ranging from 20 to 60%, making them suitable only for areas close to pipes and tank valves [60,61].

**Catalytic sensors** can also be used, but only in areas where air–hydrogen mixtures are likely to occur, such as during tank ventilation [64].

**Palladium sensors** are still the most sensitive and specific to hydrogen gas compared with other types of sensor [64].

**Optical sensors** are the preferred choice for aircraft and trains, while **palladium-based optical sensors** offer promising results in complex and critical environments [60,61,64].

According to the research carried out, the use of these sensors in anaerobic environments remains very poorly documented, with few concrete applications and few articles published on the subject, which shows that this is an area that still needs to be developed.

### 2.4. Aerobie Applications

Hydrogen gas is present in many potential applications in an aerobic environment, i.e., in the presence of oxygen and humidity, and taking into account the environment and interferents. Aerobic applications are more numerous and more in tune with current events and the energy transition, since hydrogen will be in contact with different environments and with ambient air containing 20% oxygen. In the hydrogen context, an aerobic environment could be used for leak detection and fuel cells, as well as electrolyzers and bioreactors [5,21].

#### 2.4.1. Leak Detection

Hydrogen leaks can occur at various stages of the hydrogen value chain, including production, storage, transport, and use. Compliance with hydrogen-related standards and regulations is essential when using sensors to detect hydrogen leaks, be they primary, secondary, classified by level of danger, or tertiary [33]. Table 5 shows the nature of these leaks and their hydrogen concentration ranges.

The recent paper of Mohammed W. and Zekai Hong presents hydrogen sensors that can be used for leak detection, assessing performance by sensor type, including selectivity, sensitivity, response time, detection ranges, detection limits, market position, and lifetime [65].

To be effective, hydrogen sensors must at least guarantee detection in the range of 0% to 0.1% H_2_, in order to detect early leaks and prevent the concentration from reaching the lower explosive limit, i.e., 4% [65]. The oxygen level in the event of a hydrogen leak must always be less than 19.5%, according to the Occupational Safety and Health Administration (OHSA) [22,28]. Values corresponding to current regulations can be found in Table 6. Environmental temperatures range from −30 °C to 80 °C, and relative humidity from 10% to 98%. Response times must be less than one second, and a service life of at least 10 years must be guaranteed. Each sensor must ensure sensitivity and selectivity to hydrogen, notably by resisting potential interferents such as hydrocarbons [65]. It should be noted that the sensors, whatever the context, must withstand 1 bar of pressure [65].

**Electrochemical sensors** (0.01% H_2_) are the most widely used because they achieve better leak detection performance, with high sensitivity (low/high concentrations) and a fast response time (less than one minute), despite regular maintenance and sensitivity to interferents [66].

**Both catalytic and resistance-based sensors** have lower environmental sensitivity and a service life that can be less than ten years [33,65].

**Working function sensors** (sensors with a detection function and an integrated signal processing function (filtering, amplification, self-calibration, etc.)) and **optical sensors** are expensive, limiting their use in industry, despite their optimum performance [67].

**Thermal conductivity sensors,** despite their simplicity, robustness, and low cost, have limited sensitivity to low concentrations [33,67].

For **leak detection in natural cavity storage**, which is also an important issue in the energy transition and a worldwide concern, five types of sensors have produced satisfactory performance results: electrochemical sensors, thermal sensors, palladium-effect sensors, semiconductor sensors, and optical spectroscopy sensors [68,69].

All known detection limit values for this type of application, arranged by sensor technology, are listed in Table 7.

In the field of leak detection, a number of hypotheses can be made regarding the use of sensors, depending on the type of leak, the associated ranges, and detection limits. Table 8 shows the sensors recommended for a specific type of leak.

#### 2.4.2. Fuel Cells

The fuel cell uses a redox reaction to convert the chemical energy contained in hydrogen into electrical energy. The end products of this reaction are electricity, water, and heat, with no emissions of carbon dioxide or polluting particles [39]. According to the application, sensors must be able to detect hydrogen at atmospheric pressure up to an average of 30 bar, at high humidity ranging from 80 to 100%, and at a wide range of hydrogen concentrations up to 100% [70].

Electrochemical sensors (0.01% H_2_) are highly sensitive in monitoring hydrogen, as are palladium sensors (0.001% to 0.01% H_2_), which are particularly sensitive to low levels of hydrogen [70,71]. Solid-state sensors (0.1% H_2_) are affordable and useful for monitoring large areas, while thermal sensors are used for the continuous monitoring of reactant levels in the cell and can detect an average of 0.1% to 0.5% hydrogen.

Spectroscopic sensors are often used in critical environments as they present with high accuracy (0.001% to 0.01% H_2_) [70,71,72]. Electrochemical, palladium-based metal film, and MEMS sensors can be used to detect micro-leaks and for early detection, particularly with semiconductor sensors [70,71,72]. To ensure optimum monitoring in line with safety standards, infrared or catalytic sensors will be used, or even laser diode sensors for the emergency monitoring of high concentrations [70,71,72].

#### 2.4.3. Bioreactors–Electrolyzers–White Hydrogen Research

**Hydrogen bioreactors** operate according to specific biological processes in which microorganisms generate hydrogen in controlled environments, such as dark fermentation, hydrogen photoproduction and bioelectrolysis [73,74,75]. The benefits are production from renewable resources (organic waste, biomass, water), waste reduction and CO_2_-free hydrogen production as part of the circular economy and energy transition. The development of these large-scale systems could transform waste management and the production of green hydrogen. Sensors measure hydrogen concentration in the bioreactor in real time, guaranteeing precise monitoring of the biological process, system safety, and production control [75].

**Hydrogen electrolyzers** [76] are devices that produce hydrogen by breaking down water (H_2_O) into hydrogen (H_2_) and oxygen (O_2_) using an electric current. This method is considered one of the cleanest for producing hydrogen, especially when powered generally by renewable energy. Sensors measure the concentration of hydrogen produced by the electrolysis of water, detect any leaks, and ensure that the plant is operating correctly [77].

Hydrogen sensors for bioreactors and electrolyzers [73,74,75,76,77] generally operate with detection thresholds from 0% to 100%, and alarm thresholds set at around 1–2% to prevent risks. Automated safety and control systems are essential to ensure their safe and efficient operation. Hydrogen concentration ranges from 10% to 60%, depending on the type of microorganism and substrates used [76,78]. However, the sensors must detect hydrogen concentrations of less than 1% for these applications, ensuring full resistance at a minimum of 30 bar pressure, under high humidity of around 80%, and also taking into account the presence of interfering gases. Sensors operating solely under atmospheric pressure are not suited for these applications [74]. In bioreactors [73] and electrolyzers [76,78], the types of sensors used are chosen according to the target application and the environment.

The search for white dihydrogen (H_2_ white) [59] is an approach aimed at recovering hydrogen naturally present in the ground. It is produced geologically by reactions such as serpentinization (ultrabasic water–rock interaction), radiolysis of water, or deep degassing. Its extraction without carbon-based chemical transformation and purification can have a reduced or non-zero carbon footprint, guaranteeing a low environmental impact. To detect and quantify natural H_2_ leaks into the ground and air, several types of hydrogen sensors are used. These sensors can identify areas where hydrogen naturally escapes from the subsurface and detect low concentrations of H_2_, in the ppm range. They provide data for choosing the best drilling locations, reducing exploration risks and costs, or prioritizing high-potential sites. The main objective remains to manage the risks associated with leakage. The sensors used for this type of application are similar in every respect to those used for the electrolyser application, since the search for white dihydrogen is based on electrolysis with renewable energies [59].

A summary of the detection limits in these areas is presented in Table 9 [59].

**Electrochemical sensors** with platinum electrodes are preferred because of their high sensitivity and selectivity to hydrogen, despite their limited service life and need for regular maintenance. They monitor hydrogen levels to adjust operating conditions, and operate under high humidity [43,79].

**Semiconductor sensors** are useful for monitoring the accumulation of hydrogen in the environment thanks to their sensitivity to low concentrations and their affordability. However, they are sensitive to interferents, humidity, and temperature influences, which can be detrimental, leading to loss of control in this type of application [40,44].

**Thermo-catalytic sensors** ensure reliability and robustness, but require a controlled environment where oxygen is not excessive and energy consumption is not too high [40,43,44,79].

**Palladium-based sensors** are still in the test phase [40,43,44,79]. They are compact, robust in delicate environments, specific, and selective of hydrogen, but are sensitive to interferents and surface contamination by impurities including sulfides and hydrocarbons.

Fiber-optic sensors are sensitive to temperature variations and interferents such as H_2_S and NH_3_. They are expensive, unaffected by electrical or magnetic interference, and suitable for complex environments [50]. They are often useful for monitoring hydrogen in environments where electrical safety is critical [43,79].

**Laser spectroscopy sensors** are recommended for electrolyzers only, and are used to monitor hydrogen leakage with precision or to adjust electrolysis yields. This technology provides high sensitivity, optimum accuracy, and long-range detection capabilities, but remains expensive [80].

#### 2.4.4. Chemical and Metallurgical Process Controls

Chemical processes transform reagents into useful molecules, while metallurgical processes transform ores into metals or alloys. They cover synthesis, smelting, extraction, purification, alloying, formation, and heat treatment, with multiple applications in industry, energy, and the environment [81]. Chemical and metallurgical process controls often involve monitoring hydrogen (H_2_) for reasons of safety, quality, or understanding chemical mechanisms [81].

The sensors used for this kind of application depend on the process; the ones that can be used are as follows [81,82,83]:

**Electrochemical sensors** provide general monitoring for safety and optimization (0.1% to 0.5% H_2_) [82].

**Semiconductor sensors** are effective for fast, continuous detection (0.5% to 1% H_2_), while **thermal sensors** are required for critical monitoring and dynamic environments (0.5% to 2% H_2_) [83].

**Spectroscopic sensors** are currently being developed, targeting an accuracy of (0.001% to 0.01% H_2_) over long distances, which is useful for monitoring low concentrations [46].

**Palladium-effect and spectroscopic sensors** [81,82,83] are often used for low-level detection in specific processes. Hydrogen concentration ranges vary according to the process, but sensors must guarantee functional detection under pressure (approx. 30 bar) and relative humidity ranging from 20 to 80%.

#### 2.4.5. Wastewater Treatment

Hydrogen wastewater treatment is an innovative technology for purifying and recovering hydrogen from wastewater. The process uses chemical transformation technologies to extract, treat, and use the hydrogen generated as an energy resource or as a carrier in various industrial systems. The principle of wastewater treatment with hydrogen is based on the decomposition or conversion of organic compounds contained in wastewater to generate hydrogen [72].

In this field of application, the sensors must detect low concentration ranges from 0 to 0.01% dihydrogen, under atmospheric pressure and a relative humidity of up to 80% [72]. Digestion requires sensors to detect ranges from 10 to 100 ppm, and standards for biological treatment systems require a minimum of 0.5 ppm to 5 ppm. For effluents after treatment, the range is between 0.1 and 10 ppm [72].

**Electrochemical sensors** are used to accurately measure hydrogen concentration in wastewater in real time (approx. 0.01 to 0.5 ppm) [75].

**Thermal sensors** are useful for monitoring hydrogen production by bacteria in biological treatment systems (approx. 0.1 ppm) [75].

**Semiconductor sensors** are used to monitor hydrogen generated in digestion or sludge treatment processes (approx. 0.1 to 0.5 ppm) [84,85].

**Metal film sensors** are used for the lowest concentration values, as they are sensitive and well suited to monitoring bacterial or chemical hydrogen production in wastewater (approx. 0.01 ppm to 0.1 ppm) [84,85].

**Spectroscopic sensors,** which are also highly sensitive, can detect low levels of hydrogen in wastewater (around 0.01 ppm) [75,84,85].

#### 2.4.6. Biology–Bacteriology

Hydrogen biology and bacteriology focus on the use of micro-organisms, particularly bacteria, in the production and processing of hydrogen. Its use is based on the fact that certain bacteria and micro-organisms can generate hydrogen through their metabolic reactions [86]. These reactions exploit organic substrates or light energy (photosynthesis) to convert water or organic molecules into dihydrogen. Sensors are crucial for this type of application in order to monitor hydrogen production, use bacteria as biocatalysts for hydrogen, and optimize biological processes for the creation of hydrogen with low environmental impact. Bacteria produce hydrogen through anaerobic fermentation, bacterial photosynthesis, the degradation of organic compounds, and the use of natural enzymes [86].

For this application, dihydrogen concentration ranges are very low, from 0 to 0.01% H_2_. Sensors must be able to operate at atmospheric pressure and over a wide humidity range, up to 100%. Potential interferents are mainly CO and H_2_S, but oxygen and carbon dioxide are interfering gases that can be used in several sub-applications [86]. The average values for the detection of biological fluids and bacterial production in aerobic environments are low, ranging from 0.1 to 1 ppm, while the average values for bacterial production in anaerobic environments aimed at bacterial metabolism range from 10 to 100 ppm [86].

**Electrochemical sensors** (approx. 0.01 to 1 ppm) are used to measure hydrogen concentrations in biological fluids such as plasma, serum, or exhaled air [6,87].

**Thermal sensors** are used because they are sensitive to low levels of hydrogen in biological environments (around 0.1 ppm) [6,87].

**Semiconductor sensors** can be used to monitor bacterial hydrogen production or its presence in various biological environments, with detection limits ranging from 0.1 to 0.5 ppm [6,87].

**Palladium sensors** are widely used in bacteriology to study hydrogen production by bacteria, as they detect around 0.01 ppm to 0.1 ppm [6,87].

**Spectroscopic sensors** are still being developed to detect concentrations below 0.01 ppm [6,87].

#### 2.4.7. Healthcare–Pharmaceutical Industry

The use of hydrogen in healthcare is growing thanks to its antioxidant and anti-inflammatory capacities, and its potential therapeutic effects. Hydrogen is increasingly being studied for applications in the treatment of various pathologies, as well as in the monitoring of biomarkers in medical environments using specific sensors [63,88]. Its antioxidant and anti-inflammatory properties provide protection against cell damage, leading to preventive or curative therapies. Its many applications include the prevention of heart attacks and hypertension and neurological diseases (stroke, Alzheimer’s), diabetes therapies, chemotherapy, and radiotherapy [63,88].

Sensors used for this type of application must detect low dihydrogen concentration ranges, ranging from 0 to 0.01%, and must ensure optimum detection under atmospheric pressure, taking into account a wide humidity range of up to 100% [63,88].

For hydrogen detection in blood, for example, concentrations range from 0.5 ppm to 1 ppm. These sensors’ use in exhaled air and detection in biological fluids ranges from 0.1 ppm to 0.5 ppm [89].

**Electrochemical and metal film sensors** are used because they can detect around 0.01 ppm to 0.1 ppm of hydrogen in biological environments [88,90].

**Thermal sensors** sensitive to small variations in dihydrogen concentrations in biological environments detect about 0.1 ppm, while **semiconductor sensors** detect about 0.1 ppm to 0.5 ppm [91].

**Spectroscopic sensors** enable low detection rates of less than 0.01 ppm. They are also widely used in medical diagnostics, as they enable the real-time detection of hydrogen and detect low concentrations of dihydrogen [88,90,91].

### 2.5. Influence of Oxygen on Hydrogen Sensors

In an aerobic environment, two physical phenomena occur. The oxygen present in the atmosphere is adsorbed onto the surface of the sensor. It can occupy the active sites of the sensor, preventing hydrogen from binding properly, which reduces the sensor’s sensitivity. Oxygen can also react with hydrogen to form water on the sensor surface [71,83]. This leads to a change in the electrical response and a disruption of hydrogen detection.

Platinum-based catalytic sensors, for example, detect hydrogen through a catalytic reaction. However, in the presence of oxygen, the reaction on the platinum surface produces water, which alters the sensor’s response. This can lead to reduced sensitivity and a nonlinear response, as water formation becomes the primary detection mechanism [31,92]. Semiconductor-based sensors, such as WO_3_, exhibit sensitivity to humidity. Water vapor can interact with the active sites on the surface, altering the conduction properties and affecting the sensor’s response. The addition of complementary materials capable of interacting with water molecules can improve stability under humid conditions [93].

**Figure 11:**
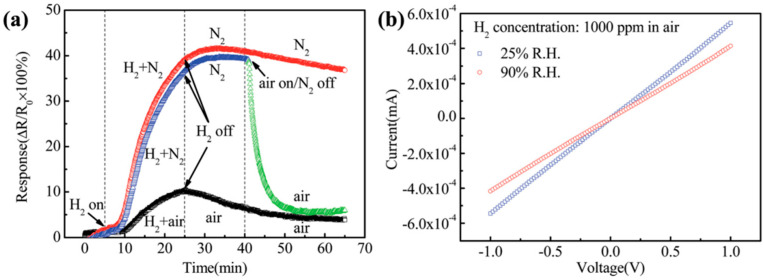
Sensor responses to 1000 ppm of hydrogen for various types of gas mixtures (**a**). I–V characteristics of the sensor exposed to 1000 ppm hydrogen in air under two different relative humidity levels (**b**) [94]. The color curves red, blue and green, then black, respectively represent the time dependence of the response when exposed to 1000 ppm H_2_ with different carrier gases including N_2_, air and N_2_ switched back to air as time proceeds for 35 min.

The studies by Y. Peng and J. Ye provide an example of the influence of oxygen and humidity on a reduced graphene oxide-based Pt-Pd sensor [94]. In Figure 11, a reduction in the response in the presence of oxygen, as well as the impact of humidity, can be observed. Figure 11a shows that the response amplitude under anaerobic conditions is 4000, whereas in an aerobic environment it does not exceed 1000. Figure 11b illustrates that for a concentration of 1000 ppm in air, the sensor’s response changes depending on the humidity level.

The influence of humidity on hydrogen sensors is also illustrated in other research studies, such as resistive thin-film sensors combining palladium nanoparticles with metal oxides like NiO [95] or other metals such as Au [43], as well as in optical sensors using palladium alloys with metals like cobalt, which enhance the sensor’s sensitivity and stability while reducing interference from water vapor and oxygen [96]. Pd/SnO_2_-based sensors have already been developed in the literature, as they exhibit excellent selectivity for hydrogen, even in the presence of humidity, ensuring a rapid response and mechanical stability in high-humidity environments [97]. Another sensor based on epitaxial graphene provides increased sensitivity to humidity along with a certain degree of robustness [98].

### 2.6. Synthesis

Application fields, performance requirements, measurement ranges, and associated publications are summarized in Table 10, Table 11 and Table 12 show the range of sensors available for aerobic and anaerobic applications, and the detection ranges of each technology. It can be seen that their applications in aerobic environments are more numerous than in anaerobic ones, that there are many different sensor technologies, and that their use depends on the context of the application.

Most studies, therefore, focus on aerobic environments, where the presence of oxygen promotes the absorption and redox mechanisms required for detection [99], and the economic and industrial needs are well established (hydrogen leaks, hydrogen stations, batteries, and laboratory research). Studies and applications in anaerobic environments are more limited, mainly due to the low surface reactivity in the absence of oxygen, the experimental complexity of measurements under controlled atmospheres, and the risks associated with handling hydrogen at high concentrations. These constraints reduce the reproducibility and sensitivity of conventional sensors based on metal oxides or catalytic processes [100]. Anaerobic environments are more specific (biogas, biological reactors, sealed fuel cells), generating few commercial needs and few scientific publications. Future research should focus on the development of oxygen-independent sensitive materials, such as PdAu alloys or graphene, the optimization of physical detection mechanisms (conductivity changes, diffusion) suitable for inert environments, and the design of microelectromechanical platforms ensuring safety in enclosed hydrogen-rich settings. These approaches would allow the application of hydrogen sensors to be extended to anaerobic environments. Future hydrogen applications in anaerobic settings include aerospace [101], ships [102], airplanes [103], and high-pressure tanks [104].

**Performance data for different hydrogen sensors:**

The sensors’ performances are often interrelated, encompassing reliable detection at both low and high hydrogen concentrations, response and recovery times of less than one minute, and long operational lifetimes [14,37,52]. Research studies in which these performances are quantified are summarized in Table 13, along with the corresponding performance metrics and references.

Table 13 shows that the lowest detection limits are achieved with optical and graphene-based nanostructured sensors. The fastest response times are observed for MEMS and plasmonic sensors. Lifetimes vary greatly depending on the sensor technology and the environment (temperature and humidity conditions). Semiconductor sensors remain the most robust and cost-effective but require high operating temperatures. Palladium-based sensors suffer from limited lifetimes and degradation due to hydrogen absorption hysteresis. Optical fiber and nanostructured technologies, such as MEMS and graphene-based sensors, enable low detection limits (below ppm and even ppb), with response times of few seconds.

Response and recovery times are key performance indicators for hydrogen sensors, mainly for applications including security, leak detection, and real-time monitoring. Xing’s work focuses on a sensor based on a homogeneous SnO_2_ composite enriched with oxygen vacancies, designed to achieve rapid response and recovery. A well-engineered material reduces response times to approximately 1.1 s and recovery times to approximately 1.9 s for In_2_O_3_–SnO_2_, representing reductions of 11% and 9.5%, respectively, compared to pure SnO_2_ [111]. Chen’s work shows that using an MXene/SnO_2_ heterojunction sensor enabled a rapid response (value unspecified). A shorter response time is obtained compared with SnO_2_ alone [112]. A PdO-modified heterojunction hydrogen sensor developed by Xing presents response and recovery times of approximately 0.8 s at 50 ppm hydrogen [113]. Yang developed an ultra-fast H_2_ detection system comprising a vertical thermal conduction structure and a neural network algorithm [114]. The response time obtained is around 0.4 s.

## 3. Hydrogen Sensors Coupled with Artificial Intelligence

Artificial intelligence (AI) is used in hydrogen sensors to **improve performance** (accuracy, sensitivity, selectivity, response time, repeatability, and drift phenomena [44]) and **sensors design**. It also involves the fusion of data from several sensors, combining several data sources to assess the presence of hydrogen [115]. In the predictive maintenance domain, algorithms are used to analyze trends in sensor measurements [116] and to optimize energy consumption [117]. In the literature, a small number of papers deal with smart hydrogen sensors, and most of them concern resistive sensors. Nevertheless, this is a recent and fast-growing field with promising results.

### 3.1. Hydrogen Sensor Design Improvement

**Figure 12:**
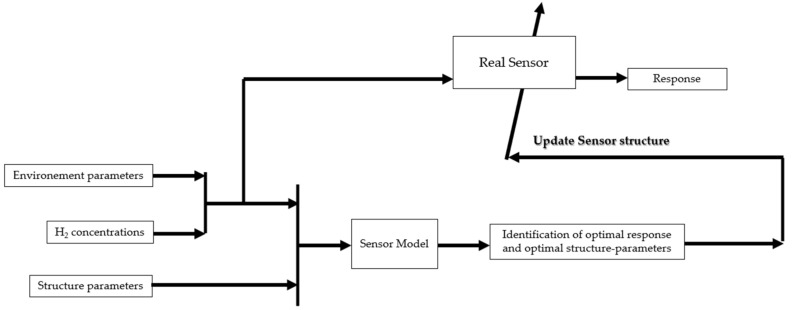
Improving sensor design from a physical point of view through a software layer.

To the best of our knowledge, there are few works that use a software layer to improve the design of hydrogen sensors from a materials point of view. This improvement can be performed using data-driven methods according to the schema shown in Figure 12. An example of improving the design of SnO_2_-based hydrogen sensors is presented below [11].

Cheng Shi and al. [11] propose a technique for predicting SnO_2_-based hydrogen gas detection performance as a function of the environment and a set of sensor structure parameters using artificial intelligence.

This technique can simulate the effects of temperature, hydrogen concentration, dopant material type, doping concentration, and other factors on material sensing performance. The idea is to use machine learning tools to build a model that describes the inherent relationship between the dihydrogen sensing response (HDR) of SnO_2_-based sensors and its corresponding influential characteristics (nano-composite chemistry and operating conditions) as follows [118]:

HDR = ML(Dopant molecular mass and dosage, temperature, H_2_ concentration) (1)

The model is used as a simulator to analyze the influence of each parameter on the response. This avoids the need for experimentation, which requires considerable resources.

The data used to train the model are summarized in Table 14.

**Figure 13:**
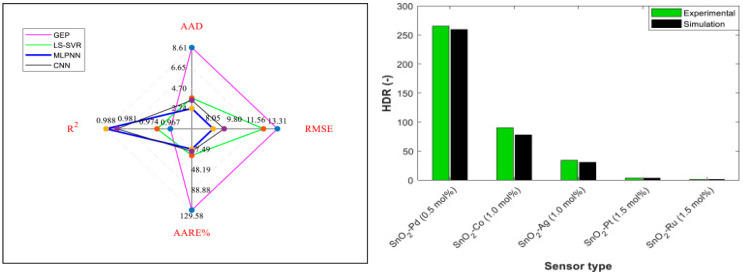
Spider plot (**left**). Evaluation of the hydrogen sensing response of different SnO_2_-based composites from experimental and modeling perspectives (**right**).

Several models have been implemented: Gene Expression Programming (GEP) [122]; Support Vector Least Squares Regression (LS-SVR) [123]; Multilayer Perceptron Neural Networks (MLPNN) [124]; and cascade (CNN) [125]. A comparative study of these models, illustrated by the spider graph in Figure 13, shows that MLPNN performs best (regression coefficient = 0.9882; mean absolute deviation = 2.74; and root mean square error = 8.05).

Trend analyses were carried out by studying the influence of dopant type on sensor HDR. The experimentally measured HDRs of the five SnO_2_-based sensors studied and their corresponding MLPNN predictions under the same operating conditions (T = 300 °C; H_2_ concentration = 1000 ppm) are plotted in Figure 13.

Firstly, acceptable agreement between the actual and predicted HDR values can be observed. Furthermore, the figure confirms that SnO_2_-based nanocomposites decorated with Pd and Ru metals exhibit the maximum and minimum sensitivity to hydrogen gas detection in air, respectively. This is an important finding for applications that require a suitable agent to decorate SnO_2_ nanoparticles to improve hydrogen detection performance.

### 3.2. Improving the Performance of Hydrogen Sensors

**Figure 14:**
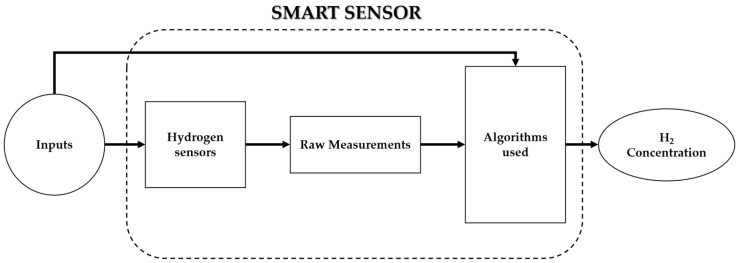
Software layer to enhance sensor performance.

Artificial intelligence is increasingly used to improve the performance of hydrogen sensors. Performance targets include accuracy, sensitivity, selectivity, response time, repeatability, and the phenomenon of drift caused by the aging of hydrogen sensors. Most of the research work found in the literature concerns resistive sensors that integrate the software part downstream of the raw sensor measurement, as illustrated in Figure 14. Depending on the target, data-driven methods are used to build classification and/or regression models.

The inputs/outputs of the algorithm depend on those of the sensor, and therefore on its technology. For all resistive sensors, the inputs are variations in resistance (∆R), voltage (T), current (I), operating temperature (T_f_), and heating temperature (T_c_), as well as other environmental variables such as relative humidity (RH), flow rate (D), and pressure (P) when measured. The outputs of the system are mainly the presence or absence of hydrogen and the evolution of its concentration. The inputs and outputs of the software layer according to the type of sensor are presented in Table 15.

**Figure 15:**
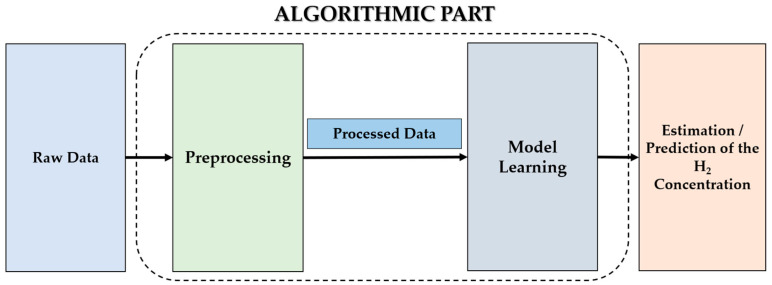
Algorithmic part.

The software part is composed of two parts, as illustrated in Figure 15. The pre-processing step is used to extract relevant characteristics of the target, followed by two types of models: classification models to detect hydrogen in a mixture, and regression models to estimate and predict its concentration.

**Figure 16:**
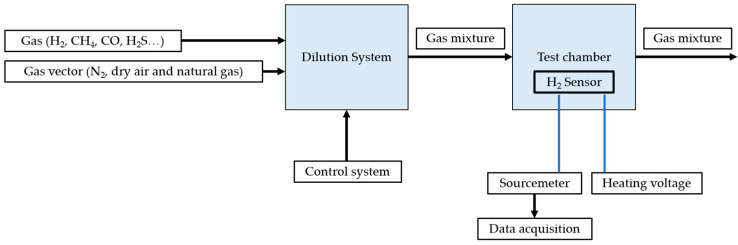
Hydrogen electrical characterization bench [42,45].

To build models, datasets are required. Test benches, providing the possibility of imposing concentrations and recording measurements of other variables, are used to build labeled matrices. Figure 16 shows a schematic diagram of a test bench used to characterize resistive hydrogen sensors.

This consists of gas sources for hydrogen, carrier gas, and interfering gases. Concentrations are regulated by a control system (mass flow) in a dilution chamber, then transferred to a gas enclosure. The sensor is connected to a Keithley 2450 type sourcemeter associated with a data acquisition and recording interface [45].

The existing research work on smart hydrogen sensors is summarized in Table 15, where sensor types, inputs, outputs, initial performance, methods and algorithms used, and final performance achieved are presented.

From Table 15, it can be seen that model inputs and outputs are defined according to the target and sensor type. Hydrogen concentration is common to all models, even when the target is to detect the presence of hydrogen.

Table 15 shows that metal oxide sensors based on various materials, such as ZnO [15], doped with Pd [155,156,157,158] or decorated with noble metals [128], the MoO_3_ [129], the SnO_2_ doped with Pd [14], the WO_3_ [159] doped with Cu, Fe, and Pt [160,161,162], or graphene [16,163], are the most frequently used.

Model inputs are generally related: resistance, self-biasing voltage, current, heating temperature, operating temperature, relative humidity when measured [143], pressure [151], and wavelength [148,149]. They depend on the type of sensor. MOX sensors use current, resistance, voltage and heating temperature, and operating temperatures values as inputs in most applications [31,142]. The same applies to electrochemical, thermal, and catalytic sensors, with relative humidity added as one of the inputs to the models [53,144]. Fiber optic sensors have specific inputs such as the reflected or transmitted wavelength of the light source or absorption spectra [47]. Other [150,152,153,154] semiconductor sensors use raster images as inputs, with each pixel corresponding to the normalized resistance [14,132,133].

In the Model Learning section, most of the existing models were tested and implemented in order to assess which ones deliver the best performance. In light of this research, several recent models offer optimal performance after the addition of a software layer. A presentation of these models of different formalisms and a performance comparison is illustrated below:

- Support Vector Machines (SVM) [164] are based on separation plane calculations [165,166]. They can be used to classify hydrogen concentration levels or perform complex regressions [167]. SVMs are used for classification problems with a nonlinear feature space in [168,169]. In [126,127], SVMs achieve 96% performance for hydrogen selectivity.
- Random Forests (RF) are also known as decision trees [170] or regression trees based on Bayes’ theory. They are used in [137,138] to improve the selectivity and accuracy of hydrogen sensors, with an accuracy of over then 90%.
- SVMs and RFs are powerful tools for regression and classification. When classes overlap and are not physically separable (by hyperplanes), the RF algorithm is more appropriate as it is based on a probabilistic decision process [171,172]. If the classes are separable, even when the separating hyperplanes are strongly nonlinear, an SVM, thanks to the multitude of kernels and kernel combinations, manages to compute the separating hyperplanes [167,169,173].
- Linear and Logistic Regression [165,166,174] is used in [175] to model the relationship between measured resistance and hydrogen gas concentration and to predict hydrogen concentration levels down to around 10 ppm.
- Long Short Time Memory (LSTM) [175,176] is a memory neural network used to predict the evolution of hydrogen concentration levels over time. This is implemented in [66,130] for concentration predictions, with a sensitivity of around 3 ppm.
- Convolutional Neural Networks (CNNs) [165,166] combines feature extraction and model learning. Inputs are raster images where each pixel corresponds to the normalized resistance obtained by an optical sensor [14,132,133]; CNNs [174,177] are frequently used for classification [178] and lead to optimum performance (almost 100%) for the identification of hydrogen among CO, NH_3_, or H_2_S [14]. Although used for image analysis, CNNs can also be applied to time series and sensor signal data, since they can extract important features from resistance signals such as patterns or trends, and thus improve the accuracy of predictions [179].
- K-Nearest Neighbors (KNN) is a classification algorithm [174] based on a distance calculation [103] and is chosen to identify low hydrogen concentrations at high noise levels, with 91% accuracy [134,135,136].
- Multi-Layer Perceptron (MLP) [180,181] is also used to differentiate between low and high hydrogen concentrations in [143].
- Other types of artificial neural networks (RNN, RNA, and ANN) [182,183] are implemented to establish complex data relationships between resistance and gas concentrations [184]. In recent research work, they are used to estimate and predict physical parameters related to hydrogen safety [151].
- Polynomial regression is an identification model used in [185,186] l for the calibration of hydrogen sensors.
- The autoencoder method, based on the statistical and variational properties of data [187], is used to detect hydrogen leaks in the presence of potential drifts in sensor response with 98% accuracy [139,140].
- Continuous Markov models are stochastic models that can be used to estimate hydrogen concentrations in the presence of uncertainties [188].
- Recent work in the IM2NP laboratory has enabled us to study the prediction of hydrogen content in natural gas pipelines supplying industrial machinery, in the context of power to gas technology, using geometric models associated with a PdAu alloy resistive hydrogen sensor, providing results for hydrogen detection, with promising performance in terms of selectivity, stability, and sensitivity [45].

All the models used guarantee good performance, with over 85% accuracy in the majority of these research projects.

However, for data preprocessing, the predominant method used is Principal Component Analysis (PCA) [189]. It was chosen because it is a highly efficient linear method for reducing data dimensionality and extracting useful features [143]. This is useful when the number of sensors or variables is high [189]. The works of Gao [143] show that PCA led to a 91% improvement in the recognition of H_2_ concentrations. PCA is used in [134,135] for hydrogen detection, with 92% accuracy.

Nevertheless, there are many other methods of feature extraction and selection in the literature [190,191], adapted to different types of sensor data, which can be used for improving the performances of hydrogen sensors.

Existing feature selection techniques can be divided into three main families: filter methods, Wrapper methods, and integrated methods [192]:

- Filtering methods: These methods are not based on the classification/regression model. They evaluate each feature independently, usually using statistical measures or correlation coefficients with the target variable. Consequently, model training and performance evaluation take place after the relevant features have been selected. They do not take into account the impact of each feature on the others, but simply evaluate them individually [193,194,195].
- Wrapper methods: These methods select features according to their impact on model performance. The specific machine learning algorithm is used to evaluate the effect of each feature or group of features on model performance. Features are retained or removed according to their ability to improve model performance. This technique is distinguished by its accuracy and its ability to take into account interactions between features [195,196].
- Integrated methods: These methods enable features to be selected directly during model training. Each model or algorithm selects the most important features according to the mechanism used during training (coefficient evaluation, data slicing, etc.). Unlike the Wrapper method, which involves training the model on selected features and then evaluating its performance, the Embedded technique stands out for its speed and high accuracy. However, it may not always achieve the same level of accuracy as the Wrapper method [195,197]. The CNN used in [14,19,132,133] is one of the methods that integrates the extraction/selection step through its convolutional layer.

For model learning, most of the data comes from labeled matrices, which are mostly built on experimental test benches. For example, the research by Ali Salimian [19] was carried out using an in-house laboratory test bench with an integrated spectrometer for hydrogen detection. The data was then recovered and used to train regression or classification models, depending on the type of problem. The same applies to most of the work in Table 15, such as that by Liu [47] or Sutarya [151]. Sometimes, for certain projects, data not integrated into the bench but retrieved from other in-house sensors were used for the software, as in the work of Diao [132] and Sun [133], with optical sensors being used to obtain images of resistance variations; from Gao [143] with a relative humidity sensor; and from Thanh [146] and Gardner [147], using a manometer-type sensor to measure pressure.

All this data provides the input for the algorithmic part [190,191], including data pre-processing [174,198,199] and a model learning section [165,166,200].

The performance of a model built with laboratory data may vary in a real environment, as real environmental conditions remain more complex than a controlled laboratory environment.

Other types of sensors have not been associated with a software layer. To our knowledge, there is no combined network of hydrogen sensors in different materials coupled with AI tools. Similarly, work on **thermal and electrochemical sensors** shows that there are currently no sensors of this type on the market that operate with a library of models [201,202]. For **optical and spectroscopic sensors**, coupling to AI is necessary to improve selectivity, precision, and spectral resolution, or to compensate for drift or environmental effects, such as temperature and humidity, and to detect micro-leaks [49,203,204]. **However, these types of sensors are aimed at industry and are designed by international manufacturers.** They are equipped with a software layer, but little research has been carried out in this area for fiber-optic and spectroscopic hydrogen sensors [67,149,205].

### 3.3. Artificial Intelligence in Gas Detection

Regarding the general case of gas sensors associated with AI, numerous studies have been reported. Zhang proposes a gas sensor network combining a carbon-based thin-film transistor and an LDA-LR (logistic regression) algorithm for the detection and identification of harmful indoor gases [206].

Claudia Gonzalez Viejo et al. employed two artificial neural network (ANN) to predict the peak areas of 17 different volatile aromatic compounds and the intensity of 10 sensory descriptors regarding the quality of beer [207]. Similarly, an electronic nose was designed to identify the baking level of cookies and classify them into specific categories using Convolutional Neural Networks (CNN) [208].

AI is also emerging in the field of healthcare; sensors contribute to the detection of key breath gases such as acetone, ammonia, hydrogen sulfide, and nitric oxide for real-time health monitoring. The need to improve the selectivity of respiratory sensors is addressed through the integration of machine learning (ML) algorithms, including convolutional neural networks (CNN) and support vector machines (SVM) [164].

Recent work within IM2NP-Lab [172,209] focuses on the detection and estimation of ethanol concentration in a disturbed environment using MOX sensors and the Random Forest (RF) method. The results demonstrate that the RF model achieves an accuracy of 94%.

## 4. Conclusions

Today, the development of hydrogen sensors is an attractive challenge in the context of energy transition. Indeed, a wide variety of applications require hydrogen detection or measurement systems due to safety, monitoring, and maintenance issues. The device must be low-cost, selective, and sensitive to hydrogen, reproducible over a wide humidity range, and able to operate at atmospheric pressure or under pressure under low- and high-hydrogen concentrations, and taking into account physical disturbances, the environment, the application, and the presence of interfering gases. For this reason, several types of sensors are being developed, which are increasingly associated with Artificial Intelligence.

This paper provides an overview of hydrogen applications in anaerobic and aerobic environments. It highlights the available sensor technologies, with detection ranges specific to each device. All the applications discussed in this review are associated with sensors of various types. This review assesses and discusses their compatibility with each application, while highlighting their measurement ranges.

The use of a software layer to improve the structure or performance of hydrogen sensors is a growing area of research. In this paper, an overview of the methods used and the performance achieved is presented.

Most existing models of classification and regression were studied, from the simplest, such as polynomial models, to the most recent, such as convolutional neural networks and autoencoders. However, the pre-processing part is limited to the use of PCA. In this paper, a summary of existing feature selection methods is proposed to guide future research in this field.

The software layer of the intelligent hydrogen sensors studied in this paper is often built using labeled databases derived from test scenarios carried out in the laboratory in a controlled environment. Few studies have looked at the evolution of the performance obtained in the laboratory during tests in real environments.

## Figures and Tables

**Table 2:** Specification requirements [52].

Specifications Requirements	Catalytic	Electrochemical	MOX	Thermal Conductivity	Metal Films
**Anaerobic**	✗	✓	✗	✓	✓
**Dry environment**	✓	✗	✓	✓	✓
**Temperature < 100 °C**	✗	✓	✗	✓	✓
**Measurement 0–100% H_2_**	✗	✓	✗	✓	✓
**Selective to H_2_**	✗	✓	✗	✗	✓
**T_90_/T_10_ < 1 min**	✓	✓	✓	✓	✓

**Table 3:** Ranges according to vehicle type and benefits [61].

Vehicle Types	Sensitivity	Advantages
Planes	10 ppm to 4%	Priority given to rapid detection to avoid any risk in flight
Trains and buses	100 ppm to 4%	Robustness against vibrations and interference
Vehicles	100 ppm to 4%	Compact and low cost
High pressure tanks	10 ppm to 100%	Low leakage than hydrogen-saturated environments

**Table 4:** Detection ranges by sensor technology [60,61].

Sensors	Measurement Ranges	Advantages
Semiconductors (SnO_2_, ZnO)	1 ppm to 1%	Sensitive to low concentrations
Electrochemical (Pt, Pd, Au, Ni or Co)	10 ppm to 10%	Accurate and reliable for critical applications
A palladium	1 ppm to 100%	Very sensitive and specific to hydrogen
Optics (Pd)	10 ppm to 100%	Demanding environments (airplanes, trains)

**Table 5:** Type of leak and associated concentration ranges [33].

Types of Leaks	Nature	Concentration Range H_2_
Primary	Trace–Background Leak	<10 ppm
Early	Beginning of leak	10–100 ppm
Secondary	High risk	100 ppm–1%
Tertiary	Immediate danger	1–100%

**Table 6:** The values of OSHA/INRS regulations [22,23,24,26,28,29].

Settings	Concentration (in % by Volume)
Short-term exposure threshold	1% for 15 min
Average exposure threshold	1% over an 8 h period
Minimum flammability limit	4%
Alarm threshold in natural cavities	1% to 2%
Average concentration monitored for safety in the natural environment	0.1% to 2%

**Table 7:** Detection limit values for different technologies [68,69].

Technology	Detection Limit Value (in % by Volume) of H_2_
Electrochemical	0.01%
Semiconductors	0.1%
Thermals	0.1% to 0.5%
Palladium effect	0.001% to 0.01%
Spectroscopiques	0.001% to 0.01%

**Table 8:** Recommended sensors depending on the nature of the leak.

Nature of the Leak	Recommended Sensors	Advantages
Slow leak at very low H_2_ concentration	Electrochemical or MOX sensor	High sensitivity (ppm); low cost; use for environmental monitoring
Rapid leak at moderate H_2_ concentration	Catalytic sensor or thermal sensor	Detection around 4% H_2_–Fast response
Continuous leak in controlled atmosphere	Optical sensor or Pd-based metal film sensor	Stable; reliable; little sensitive to external interference
Significant leak with high risk of explosion (ATEX zones)	Catalytic sensor	Robust and suitable for complex environments
Leaks in environments with multiple gases	Fiber-optic sensor or infrared spectroscopy sensor	High selectivity to H_2_; little cross-interference

**Table 9:** Detection limit value for each technologies of sensors [59].

Sensors Technologies	Detection Limit Value (in % by Volume)
Electrochemical	0.01%
Semiconductors	0.1% to 0.5%
Thermals	0.1%
Palladium effect	0.001% to 0.01%
Spectroscopiques	0.001% to 0.01%

**Table 10:** Aerobic and anaerobic applications: state of the art.

Application	Range H_2_ (%)	Pressure (Bar)	Humidity (%)	Interfering Gases	References
**Anaerobic**					
Power to Gas (P2G)	1–20	30		CO–H_2_S	[42,45,48,51,52,53,54,55,56,57,58]
Monitoring of tanks of future vehicles (Train/Bus/Cars)	0.01–4	300–700		CO–CO_2_–O_2_–S	[59,60,61,62,63,64]
Monitoring of H_2_ aircraft tanks	0.001–4	350–700		CO–CO_2_–O_2_–S	
High pressure tank monitoring	0.001–100	200–700		CO–CO_2_–O_2_–S	
**Aerobic**					
Leak detection (safety)	0–0.1	1	20–80	CO–H_2_S–H_2_O–O_2_	[33,63,65,66,68,69]
Fuel cells	90–100	30	80–100	CO–H_2_S	[39,70,71,72]
Bioreactors–Electrolyzers	<1	30	80	CO–H_2_S–S	[40,43,44,59,73,74,75,76,77,79,80]
White H_2_ research or leak detection from storage in natural cavities	0–1	1	20–80	CO–H_2_S–H_2_O–O_2_	[59,68,69]
Chemical and metallurgical process controls	Variable depending on the process	30	Possible	CO–H_2_S	[46,81,82,83]
Pharmaceuticals	0–0.1	1	20–80	CO–H_2_S–H_2_O–O_2_	[88,90,91]
Wastewater treatment–Biology–Bacteriology	0–0.01	1	20–100	CO–H_2_S–H_2_O–O_2_	[6,72,75,84,85,86,87].
Health	0–0.01	1	20–100	CO–H_2_S–H_2_O–O_2_	[63,88]

**Table 11:** Sensor technology detection ranges for anaerobic applications.

**Anaerobic**	**Applications**	**Detection Ranges According to Sensor Types**															
		Catalytic	Electrochemical	MOX semiconductor	Thermal conductivity	Metallic films	Transistor	Optical fibers	Ultrasound	Laser Spectroscopy	Diode Laser	Infrared	Hall effect	Thermals	Spectroscopiques	Other Optics (reflectivity, transmission)	Medical diagnosis
	Power to Gas	0.1–100%	0.5 ppm–1%	1 ppm–4%	0.5–100%	1 ppm–4%	NI	NI	NI	NI	0.01–100%	NI	NI	NI	10 ppb–100%	10 ppb–100%	NI
	Monitoring the tanks of future vehicles	NI	0.5 ppm–1%	1 ppm–4%	NI	10 ppb–10%	NI	NI	NI	NI	NI	K-NI		NI	NI	NI	NI
	H_2_ Aircraft Tank Monitoring	NI	0.5 ppm–1%	1 ppm–4%	NI	10 ppb–10%	NI	NI	NI	NI	0.01–100%	NI	NI	NI	NI	NI	NI
	Monitoring of H_2_ train tanks	0.1–100%	0.5 ppm–1%	1 ppm–4%	0.5–100%	NI	NI	NI	NI	NI	NI	NI	NI	NI	NI	NI	NI
	High pressure tank monitoring	0.1–100%	0.5 ppm–1%	1 ppm–4%	0.5–100%	10 ppb–10%	NI	NI	NI	NI	0.01–100%	NI	NI	NI	10 ppb–100%	10 ppb–100%	NI

NI: Non-Identified: Values are not known or have not been specified; K-NI: Known but Non-Identified: These sensors are used but the values are not specified.

**Table 12:** Sensor technology detection ranges for aerobic applications.

**Aerobic**	**Applications**	**Detection ranges according to sensor types**															
		Catalytic	Electrochemical	MOX semiconductor	Thermal conductivity	Metallic films	Transistor	Spectroscopiques	Ultrasound	Laser Spectroscopy	Diode Laser	Infrared	Hall effect	Thermals	MEMS	Other sensors	Medical diagnosis
	Leak detection	0.1–100%	0.5 ppm–1%	1 ppm–4%	0.5–100%	10 ppb–10%	NI	10 ppb–100%	0.1–100%	0.01–100%	NI	100 ppm–100%	1 ppm–10%	0.5–100%	NI	NI	NI
	Fuel cells	0.1–100%	0.5 ppm–1%	1 ppm–4%	NI	10 ppb–10%	NI	NI	NI	NI	0.01–100%	100 ppm–100%	NI	NI	10 ppb–100%	NI	NI
	Bioreactors-Electrolyzers	0.1–100%	0.01%	0.1%	NI	10 ppm–0.01%	NI	10 ppm–0.01%	NI	0.01–100%	NI	NI	NI	0.1–0.5%	NI	NI	NI
	White H_2_ research–Detection of leaks from storage in natural cavities	0.1–100%	10 ppm–0.01%	0.01–0.1%	NI	1 ppm–0.01%	NI	0.1 ppm–0.01%	NI	0.01–100%	NI	NI	NI	0.01–0.1%	NI	NI	NI
	Chemical and metallurgical process controls	NI	0.01%	0.1%	NI	10 ppm–0.01%	NI	10 ppm–0.01%	NI	NI	NI	NI	NI	0.1–0.5%	NI	NI	NI
	Pharmaceuticals	NI	0.01–0.1 ppm	0.1 ppm	NI	0.01–0.1 ppm	NI	0.01 ppm	NI	NI	NI	NI	NI	0.05 ppm–0.1 ppm	NI	NI	NI
	Wastewater treatment	NI	0.01–0.5 ppm	0.1–0.5 ppm	NI	0.01–0.1 ppm	NI	0.01 ppm	NI	NI	NI	NI	NI	Roughly 0.1 ppm	NI	NI	NI
	Biology-Bacteriology	NI	0.01–0.1 ppm	0.1–0.5 ppm	NI	0.01–0.1 ppm	NI	<0.01 ppm	NI	NI	NI	NI	NI	Roughly 0.1 ppm	NI	NI	NI
	Health	NI	0.01–0.1 ppm	0.1–0.5 ppm	NI	0.01–0.1 ppm	NI	<0.01 ppm	NI	NI	NI	NI	NI	Roughly 0.1 ppm	NI	NI	<0.1 ppm

NI: Non-Identified: Values are not known or have not been specified; K-NI: Known but Non-Identified: These sensors are used but the values are not specified.

**Table 13:** Key performances for different hydrogen sensor technologies.

Sensor Type	Detection Limit	Response Time	Stability-Lifetime	References
Pd-based metallic film	0.1–100 ppm	1–10 s	Maximum 1 year	[99]
Semiconductor	1–100 ppm	1 min	More than 1 year	[100]
Optical fiber	10 ppb–1 ppm	1–30 s	Maximum 2 years	[105,106]
Electrochemical	0.5–10 ppm	1 min	1–3 years	[107]
MEMS	100 ppb–1 ppm	Less than 1 s	Maximum 6 months	[108]
Graphene-based	1 ppb–10 ppm	Less than 5 s	Maximum 1 year	[109]
PdAu-based plasmonic	0.1–10 ppm	Less than 2 s	Maximum 1 year	[110]

**Table 14:** Study of the chemical properties of dopants for hydrogen detection.

	Inputs				Outputs		
Sensor	Molecular Weight of the Dopant (g/mol)	Doping Dosage (mol%)	Temperature (°C)	Concentration H_2_ (ppm)	HDR (−)	Number of Data	References
SnO_2_–Ag	107.87	0–5	150–480	1.07–2000	0.6–80.1	137	[119]
SnO_2_–Co	58.93	0.196–1.195	260–400	100–35,000	1.1–291.4	51	[120]
SnO_2_–Pd	106.42	0.5	175–225	50–1000	105.7–265.5	4	[56]
SnO_2_–Pt	195.08	0.155–1.552	150–350	500–10,000	1.0–148.8	46	[121]
SnO_2_–Ru	101.7	0.298–4.408	200–350	500–10,000	1.2–26.7	45	[118]

**Table 15:** Existing research on hydrogen sensors coupled with a software layer.

Type of Hydrogen Sensors	Inputs	Outputs	Objectives	Performance Before	Methods and Algorithms Used	Performance After	References
Pd-doped ZnO nanorods	Series of resistors with derivatives and peaks	H_2_ concentration (5 ppm–500 ppm)–150–200 °C Binary classification marked by the presence or absence of H_2_	Evaluating hydrogen selectivity	75% precision	Support Vector Machines **(SVM)**	96% precision	M. Kumar [126], J. Zhu [127]
ZnO decorated with noble metals	Resistors Current Voltage	H_2_ concentration	Improving selectivity and sensitivity	Reliable detection of hydrogen; low selectivity among CO and H_2_S	Principal Component Analysis **(PCA)** + Random Forest **(RF)** Using a series of classifiers to evaluate results	Reduces classification errors by 40%	Yeong Min Kwon [128]
MoO_3_	Resistance time series over 60 sliding seconds	H_2_ concentration	Predicting H_2_ concentration levels over time	RMSE around 15 ppm	**LSTM**	RMSE reduced to 3 ppm	[66,129,130] Other studies suggest an improvement from a materials point of view [131]
Pd-doped SnO_2_	5 × 5 images where each pixel corresponds to the normalized resistance	H_2_ concentration	Evaluate the selectivity of H_2_ among CO, NH_3_ and CH_4_	55–65% correct identification of H_2_	Classification **CNN**	97% identification of H_2_	Y. Shubin [14] S. Diao [132]
Pd-doped SnO_2_	2D raster images	Binary classification marked by the presence or absence of hydrogen	Diagnosis of sensor faults	66–75% in normal and noisy environments	Method **CNN with RF**	100% in normal and noisy environments	Y.Sun [133]
Au-doped SnO_2_	Voltage Resistance Heating temperature	Low H_2_ concentration (1–100 ppm)	Identify low hydrogen concentrations at high noise levels	65% detection	**PCA** + **KNN**	92% detection	[134,135] This method can be complemented by the work of Zeng [136]
Wo_3_ doped Cu, Fe and Pt	Voltage Current Resistance variations with different gases Slope Area	Gas classification and concentration	Evaluate the selectivity of hydrogen among CO or NH_3_	60% precision	**RF**	93% precision	Yu [137] Wang [138]
Graphene/SnO_2_	Resistance values versus time Voltage Current	H_2_ concentration Reconstruction errors in order to identify the presence of an anomaly by assessing threshold exceedance	Detect potential leaks or drifts in normal sensor behavior	58% anomaly detection	Autoencoder method	98% fault detection	H. Mirzaei [139] J. Seo [140]
Catalytic	Temperature Resistance and response Voltage	H_2_ concentration	Improving hydrogen selectivity and detection	NI	Linear regression Neural network	Constant error level for all gases	D. Spirjakin [141] Ivanov [31,142]
Catalytic based PdO/SnO_2_	Relative humidity Resistance Voltage Current Heating temperature	H_2_ concentration	Identify hydrogen concentrations in a very humid environment (80% RH) Evaluate hydrogen selectivity	65–70% identification but many errors	Classification: **PCA + RF** (feature extraction and selection) **MLP** to differentiate hydrogen concentrations	91% recognition of H_2_ concentrations	Gao et al. [143]
Electrochemical-Thermal	Current Self-biasing voltage Operating temperature Relative humidity	H_2_ concentration	Improve performance in terms of selectivity and drift	10–100 ppm Not very selective Time less than 30 s Service life 1 year	**MLP** to recognize hydrogen among potential interferents + **SVM**–**RF** for multi-gas classification	10 ppm 5–20 s H_2_ detection with CO and CH_4_ Service life up to 2 years	N. Franic, I. Pivac, F. Barbir [34,53,144,145]
Thermal conductivity (MEMS)	Resistor Heating temperature Pressure	H_2_ concentration	Detection of hydrogen concentrations from 500 ppm to 3%	100 ppm for a 5 s response time	**Polynomial regression** calibration; **MLP** to compensate for temperature effect	100 ppm for a 5 s response time	H. Thanh [146] E. Gardner [147]
Fiber-optic hydrogen sensors with multilayer Pd-Y films	Wavelength of light source Absorption spectrum	H_2_ concentration	Improve sensitivity and response speed to hydrogen	Wavelength drift introducing measurement errors and influencing detection	Fast Fourier transform **(FFT)** filtering methods; moving average algorithm for spectral data analysis	Optimal design enabling a 97% reduction in measurement error Lower wavelength drift error	Y. Liu [47]
Pd-based optical fibers-Spectroscopic	Reflected or transmitted wavelength (nm) Temperature Absorption or interference spectrum sometimes	H_2_ concentration	Study drift and obtain a spatio-temporal profile when using a sensor network	NI	NI Sensors designed for industrial use by international manufacturers. Little information available on the internal software layer.	Measurements are repeatable and temperature effects are compensated from −10 to 80 °C for a detection threshold of 50 ppm	Zhang [148,149] [67,150]
Sensor for estimating and predicting hydrogen safety parameters	Heater voltage and current Heating temperature Inlet pressure and flow rate	Hydrogen outlet temperature Hydrogen outlet volumetric flow rate	Estimate hydrogen safety parameters such as pressure, temperature, and flow rates from the outlet of a Degussa sintering furnace.	NI	**RNA–ANN** Adaptative Neuro-Fuzzy Inference Systems (ANFIS) to solve a prediction problem	ANFIS >> ANN Average RMSE of 0.0387, 0.0283, 0.1301, and MAE of 0.0241, 0.0115, 0.0355 sequentially for temperature, pressure, and hydrogen flow rate	D. Sutarya [151]
Hydrogen detection using AI-based plasma discharge spectral analysis	Experimental bench with spectrometer Matrix 50 × 90 (images)	H_2_ concentration and methane	Detecting hydrogen and methane in plasma and their respective concentrations	Quantitative detection but no estimation of hydrogen content in the presence of methane	Complex **CNN** model not appropriate Residual model with predictions made after training	Not very precise (improve in terms of materials or involve more in-depth image processing)	A. Salimian [19]
Analyzers-Chromatographs	NI	NI	NI	NI	Industry-standard software such as LabVIEW	NI	[150,152,153,154]

NI: Non-Identified: Values are not known or have not been specified.

## Data Availability

No new data were created or analyzed in this study. Data sharing is not applicable to this article. The PRISMA Checklist is provided in the Supplementary Materials.

## Supplementary Materials

The following supporting information can be downloaded at: https://www.mdpi.com/article/10.3390/s25226936/s1, PRISMA 2020 Main Checklist. Reference [210] is cited in the Supplementary Materials.

## Abbreviations

AI	Artificial Intelligence
IEA	International Energy Agency
OSHA	Occupational Safety and Health Administration
INRS	National Institute for Research and Security
NIOSH	National Institute for Occupational Safety and Health
LEL	Lower Explosive Limit
UEL	Upper Explosive Limit
AEL	Average Exposure Limit
STEL	Short Term Exposure Limit
SDL	Sensor Detection Limit
HDR	Hydrogen Detection Response
ML	Machine Learning
DL	Deep Learning
MM	Mathematical Modeling
GEP	Gene Expression Program
LS-SVR	Least Squares Support Vector Regression
MLPNN	Multilayer Perceptron Neural Networks
SVM	Support Vector Machine
PCA	Principal Component Analysis
CNN	Convolutional Neural Network
ANN	Artificial Neural Network
RNN	Recurrent Neural Network
MLP	Multi-Layer Perceptron
FFT	Fast Fourier Transform
RF	Random Forest
KNN	K-nearest neighbors
LSTM	Long Short-Term Memory
